# Postoperative outcomes of mepivacaine *vs*. bupivacaine in patients undergoing total joint arthroplasty with spinal anesthesia

**DOI:** 10.1186/s42836-022-00138-3

**Published:** 2022-07-13

**Authors:** Laura A. Stock, Kevin Dennis, James H. MacDonald, Andrew J. Goins, Justin J. Turcotte, Paul J. King

**Affiliations:** Anne Arundel Medical Center, 2000 Medical Parkway, Suite 503, Annapolis, MD 21401 USA

**Keywords:** Spinal anesthesia (SA), Total joint arthroplasty (TJA), Surgical outcomes

## Abstract

**Background:**

Spinal anesthesia (SA) has been previously associated with improved outcomes after total joint arthroplasty (TJA). The purpose of this study was to compare outcomes between various local anesthetics.

**Methods:**

This was a retrospective study of 1,328 patients undergoing primary TJA with SA from September 2020–2021 at a single institution. Patients were grouped based on TKA or THA and further separated and analyzed in terms of anesthetic agents—mepivacaine (M), hyperbaric bupivacaine (HB), or isobaric bupivacaine (IB). Subgroup analysis of same-day-discharge (SDD) patients and low- (<11 mg) and high-dose bupivacaine was performed. Statistical significance was assessed at *P*<0.05.

**Results:**

Mepivacaine use was associated with younger age, lower ASAs, and lower Charlson Comorbidity Index (CCI) scores in both THAs and TKAs. Postoperatively, significant differences were found between HB, IB, and M in LOS, the first PT ambulation distance and rates of SDD, and home discharge in both THAs and TKAs. No significant differences in outcomes were observed between high- and low-dose bupivacaine in THAs or TKAs. In SDD patients, a significant difference was found only in the first 6-clicks mobility scores. After controlling for age, BMI, sex, ASA, and procedure type, mepivacaine was found to be associated with shorter LOS, increased likelihood of SDD, home discharge, POD-0 ambulation, and, further, the first ambulation distance. No significant differences were observed in 6-clicks mobility scores, urinary retention, 30-day ED returns or 30-day readmissions.

**Conclusions:**

Both bupivacaine and mepivacaine are safe and effective local anesthetics for patients undergoing TJA as evidenced by low, similar rates of urinary retention and 30-day ED returns and readmissions. Mepivacaine does appeared to facilitate early ambulation, shorter LOS and home discharge and should be considered as the local anesthetic of choice for patients undergoing rapid recovery TJA.

## Background

As the population ages, exponential growth in total joint arthroplasties (TJA) are expected. Prior studies have cited an expected 284% increase in total hip arthroplasty (THA) and a 401% increase in total knee arthroplasty (TKA) by the year 2030 [1]. In patients undergoing TJA, the use of different anesthetic techniques has also been shown to influence a variety of surgical outcomes, such as length of stay, time to ambulation, distance of ambulation, home discharge rate, and readmission or emergency department (ED) return rate [2, 3].

Pain management is a key component of postoperative care that influences the patient’s early ambulation and ability to participate in physical therapy, both of which are important predictive measures for improved patient outcomes. Early ambulation has been shown to result in shorter length of stay (LOS), lower pain levels, and lower incidence of deep venous thrombosis (DVT) and pulmonary infection [4]. Further, physical therapy intervention on postoperative day 0 has been associated with a shorter length of stay, longer ambulation distance, and greater home discharge rate [5, 6].

Anesthetic selection is a critical component of early postoperative pain management and early ambulation. Although general anesthesia is historically used in these procedures, multiple recent studies have demonstrated the benefits of regional anesthesia (RA) [7, 8]. Regional anesthesia methods include spinal blocks, epidural blocks, and peripheral nerve blocks [9]. One of the most widely used anesthesia methods for total joint procedures is spinal anesthesia. Recent studies have focused on evaluating the effectiveness of various local spinal anesthetics, primarily mepivacaine and bupivacaine, to compare their effect on postoperative outcomes following THA and TKA. Mepivacaine is a short-acting local anesthetic with an intermediate duration of action between 90 to 240 minutes, depending on dosage and administration [10, 11]. Bupivacaine is a long-acting local anesthetic with an early onset and duration of action of 2 to 5 hours [12]. However, the onset and duration may also be manipulated depending on the formulation of the bupivacaine, such as hyperbaric or isobaric. Hyperbaric bupivacaine has a density greater than cerebrospinal fluid whereas isobaric bupivacaine is equal to the density of cerebrospinal fluid [13]. This allows for a quicker onset for the hyperbaric bupivacaine and shorter duration of motor and sensory block compared to the slower onset and longer duration of action of the isobaric formulation [13]. One study found those treated with mepivacaine had earlier ambulation, earlier return to motor function, and were more likely to have the same-day discharge compared to those treated with hyperbaric or isobaric bupivacaine [2]. Another study used a double-blind, randomized clinical trial to investigate the effects of mepivacaine and bupivacaine on a patient’s return to motor function following total hip and knee arthroplasty and found a mean time of 26 minutes sooner with mepivacaine [3]. Mepivacaine has not only been shown to shorten time to ambulation and length of stay, but also has a faster neurological recovery when compared to bupivacaine [11].

The purpose of this study was to investigate the effects of spinal anesthesia on the surgical outcomes of total knee and hip arthroplasty. We compared the effects of hyperbaric bupivacaine, isobaric bupivacaine, and mepivacaine as well as high and low doses of bupivacaine on postoperative outcomes, such as length of hospital stay, ambulation on day 0, failed same day discharge attempt, first 6-clicks mobility score, the first PT ambulation distance, urinary retention, home discharge rate, and 30-day readmission or ED return rate.

## Methods

### Study population

This study was deemed institutional review board exempt by the institution’s clinical research committee. A retrospective observational study was performed in 1,328 patients who underwent primary unilateral THA or TKA with spinal anesthesia from September 2020 through September 2021. Patients undergoing bilateral or revision surgery, and those receiving general anesthesia were excluded from the study. All procedures were performed by 10 board-certified orthopedic surgeons. Procedures were performed at either an acute care hospital or an affiliated ambulatory surgery center (ASC).

### Perioperative protocol

All patients were cared for in a coordinated Joint Replacement Center and received written educational materials, a nurse taught preoperative course, preoperative medical evaluations, and preoperative strengthening programs, including home exercise or outpatient physical therapy. An established rapid recovery protocol was utilized for all patients and the protocol included a multimodal pain management regimen of celecoxib, acetaminophen, pregabalin, and short-acting opioids. Patient-controlled analgesia and nerve blocks were not used in this patient population. All patients received spinal anaesthetic agents administered into the intrathecal space via a lumbar puncture. At anesthesiologist discretion and on patient request, spinal anesthesia was paired with propofol sedation. Patients receiving spinal anesthesia were not intubated, mechanically ventilated, and did not receive inhaled anesthetic agents.

### Study design and data analysis

The patients were first separated between THA and TKA. Patient presentation and outcomes were then compared between those receiving hyperbaric bupivacaine, isobaric bupivacaine or mepivacaine. Patient demographics (age, body mass index [BMI], and sex) and comorbidity burden as measured in terms of the American Society of Anesthesiologists (ASA) score and Charlson Comorbidity Index (CCI) and were compared across groups. For each group, details of local anesthetic dosing were evaluated. Postoperative outcomes were then compared across groups. Univariate analysis, including Chi-square tests and Fisher’s Exact tests, were used to compare categorical variables. For continuous measures, two-sided independent samples *t*-tests and one-way analysis of variance (ANOVA) were conducted to compare presentation and outcomes across anesthetic types. *Post-hoc* Bonferroni adjustment was performed to make multiple comparisons across continuous endpoints. Two subgroup analyses were then performed to compare outcomes between low dose (<11 mg) and high dose (≥11 mg) bupivacaine, and between same-day discharge patients receiving either bupivacaine or mepivacaine. Finally, multivariate linear and Logistic regression models were created to evaluate the compare outcomes between patients receiving bupivacaine and mepivacaine after controlling for age, BMI, sex, ASA score, and procedure type. All statistical analyses were performed by means of SPSS (version 27.0, IBM, Armonk, NY). Statistical significance was assessed at *P*<0.05.

### Outcome measures

The following outcomes were assessed: 0-day LOS, LOS days, postoperative day 0 (POD0) ambulation, failed same day discharge (SDD) attempt, first Activity Measure for Post-Acute Care (AM-PAC) 6-Clicks mobility score, the first physical therapy (PT) documented ambulation distance, urinary retention, home discharge, 30-day readmission and 30-day ED return. POD0 ambulation included any documented ambulation by nursing or PT. The 6-Clicks mobility score is a tool used to assess a patient’s mobility and ability to complete tasks. It contains a series of questions graded on a 1 to 4 point scale, in which a physician or physical therapist completes in order to assess a patient’s progress [14]. Urinary retention was defined as any instance in which a straight or foley catheter was placed postoperatively. Readmissions and ED-returns were captured for patients returning to both the same hospital and outside hospitals.

## Results

### Demographics and comorbidities

Comparison of demographics and comorbidities between hyperbaric bupivacaine, isobaric bupivacaine, and mepivacaine found significant differences in age, ASA, and Charlson Comorbidities Index (CCI) score across groups. Both TJA cohorts showed that mepivacaine use was associated with younger patients (THA 63.2 ± 9.6, *P*=0.001; TKA 66.1 ± 8.5, *P*<0.001), ASA score of less than 3 (THA *P*<0.001; TKA *P*=0.001), and lower CCI scores (THA *P*<0.001; TKA *P*=0.004) (Table 1). The medication dosing for each of the three groups are detailed in Table 2.

**Table 1:** Patient demographics and comorbidities

	Total Hip Arthroplasty				Total Knee Arthroplasty			
Variable – *n* (%) or Avg. ± SD	Hyperbaric Bupivacaine (***n***=401)	Isobaric Bupivacaine (***n***=34)	Mepivacaine (***n***=232)	***P***-Value	Hyperbaric Bupivacaine (***n***=502)	Isobaric Bupivacaine (***n***=28)	Mepivacaine (***n***=131)	***P***-Value
Age	66.7 ± 11.4	65.3 ± 12.0	63.2 ± 9.6	**0.001**	68.9 ± 8.3	68.9 ± 8.2	66.1 ± 8.5	**<0.001**
BMI	29.4 ± 5.4	30.0 ± 6.0	28.5 ± 5.2	**0.072**	31.7 ± 5.3	31.3 ± 5.5	30.7 ± 4.9	**0.061**
Female	224 (55.9)	14 (41.2)	130 (56.0)	0.242	314 (62.5)	20 (71.4)	131 (50.0)	**0.001**
ASA ≥ 3	154 (38.4)	18 (52.9)	50 (21.6)	**<0.001**	225 (44.8)	10 (35.7)	82 (31.3)	**0.001**
CCI	3.15 ± 1.78	3.09 ± 1.73	2.48 ± 1.56	**<0.001**	3.35 ± 1.76	3.29 ± 1.27	2.91 ± 1.70	**0.004**

*P*-Values <0.05 in bold

*BMI* body mass index, *ASA* American Society of Anesthesiologists Score, *CCI* Charlson Comorbidity Index

**Table 2:** Spinal anesthetic dosing

Dose - mg	Total Hip Arthroplasty			Total Knee Arthroplasty		
	Hyperbaric Bupivacaine (***n***=401)	Isobaric Bupivacaine (***n***=34)	Mepivacaine (***n***=232)	Hyperbaric Bupivacaine (***n***=502)	Isobaric Bupivacaine (***n***=28)	Mepivacaine (***n***=131)
Mean	10.9	12.7	63.4	10.7	11.0	65.2
SD	1.7	2.1	75.3	1.6	2.4	54.5
Median	10.5	12.5	60.0	10.5	11.1	60.0
25th Percentile	10.5	11.5	56.0	9.8	10.0	60.0
75th Percentile	12.0	15.0	60.0	11.3	12.0	60.0

### Postoperative outcomes

In evaluating postoperative outcomes, patients receiving mepivacaine had the highest rate of 0-day length of stay (THA 60.8%; TKA 52.7%, both *P*<0.05) and the shortest overall length of stay (THA 0.43 ± 0.59; TKA 0.51 ± 0.59, both *P*<0.05). No statistically significant differences in rates of failed SDD attempts were observed across anesthetic groups. Mepivacaine patients were also more likely to ambulate on postoperative day 0 compared to bupivacaine for both THA and TKA (*P*<0.05). Mepivacaine and hyperbaric bupivacaine patients each had higher first 6-Clicks mobility scores compared to isobaric bupivacaine in THA but not in TKA (between groups *P*=0.003, *post hoc P*<0.05). In both the THA and TKA groups, significant differences in the first PT ambulation distance were observed across anesthetic types (both *P*<0.001), but a significant improvement in ambulation distance between the mepivacaine and other groups was observed only in patients undergoing TKA. There was no significant difference in rates of urinary retention amongst either the THA or TKA groups. Significant differences in the rate of home discharge were observed in the THA group, with mepivacaine patients being discharged home more frequently (100%) than either bupivacaine group (*P*<0.05). No significant difference in the rate of home discharge was observed in the TKA group. There was a significant difference in 30-day readmission rates for the TKA group, with patients receiving hyperbaric bupivacaine experiencing higher rates than those receiving mepivacaine (*P*<0.05). No significant differences in readmission rates were observed across anesthetic groups for THA patients, and no significant differences in 30-day ED returns were observed for either THA or TKA (Table 3).

**Table 3:** Unadjusted postoperative outcomes: all spinal anesthesia groups

Outcome – *n* (%) or Avg. ± SD	Total Hip Arthroplasty				Total Knee Arthroplasty			
	Hyperbaric Bupivacaine (***n***=401)	Isobaric Bupivacaine (***n***=34)	Mepivacaine (***n***=232)	***P***-Value	Hyperbaric Bupivacaine (***n***=502)	Isobaric Bupivacaine (***n***=28)	Mepivacaine (***n***=131)	***P***-Value
0-Day LOS	96 (23.9)a	8 (23.5)a	141 (60.8)b	**<0.001**	55 (11.0)a	4 (14.3)a	138 (52.7)b	**<0.001**
LOS Days	0.92 ± 0.79a	1.12 ± 1.00a	0.43 ± 0.59b	**<0.001**	1.09 ± 0.91a	1.00 ± 0.61a	0.51 ± 0.59b	**<0.001**
Failed SDD Attempt	22 (5.5)a	4 (11.8)a	14 (6.0)a	0.334	17 (3.4)	0 (0.0)	6 (2.3)	0.449
Ambulated POD0	282 (70.3)a	19 (55.9)a	196 (84.5)b	**<0.001**	328 (65.3)a	17 (60.7)a	225 (85.9)b	**<0.001**
First 6-Clicks Mobility Score^a^	20.48 ± 2.75a	18.82 ± 2.92b	20.43 ± 2.51a	**0.003**	20.13 ± 2.75a	20.5 ± 2.5a	19.98 ± 2.50a	0.607
First PT Ambulation Distance (ft)^b^	151.52 ± 97.51a	142.55 ± 114.21a,b	184.30 ± 84.84b	**<0.001**	134.57 ± 98.89a	111.67 ± 77.44a	165.24 ± 101.79b	**<0.001**
Urinary Retention	5 (1.2)a	2 (5.9)b	3 (1.3)a,b	0.097	6 (1.2)a	0 (0.0)a	2 (0.8)a	0.734
Home Discharge	389 (97.0)a	31 (91.2)a	232 (100.0)b	**0.001**	486 (96.8)a	27 (96.4)a,b	260 (99.2)b	0.106
30-Day Readmission	8 (2.0)a	0 (0.0)a	4 (1.7)a	0.699	30 (6.0)a	0 (0.0)a,b	4 (1.5)b	**0.008**
30-Day ED Return	15 (3.7)a	0 (0.0)a	5 (2.2)a	0.305	15 (3.0)a	0 (0.0)a	9 (3.4)a	0.599

*P*-Values <0.05 in bold

Subscript letters denote *post* *hoc* groups that do not differ from each other at *P*<0.05, continuous values are Bonferroni adjusted.

*LOS* length of stay, *SDD* same day discharge, *POD* postoperative day, *PT* physical therapy, *ED* emergency department

^a^n= THA 367 HB, 33 IB, 188 M; TKA 502 HB, 28 IB, 262 M

^b^n= 362 HB, 33 IB, 179 M; TKA 467 HB, 27 IB, 191 M

In the subgroup analysis comparing low dose (<11 mg) and high dose (≥11 mg) bupivacaine, no significant differences in any outcome measure were observed for either the THA or TKA groups (Table 4). In the second subgroup analysis comparing bupivacaine and mepivacaine in same-day discharge patients, significant difference was observed only in a higher first 6-Clicks mobility score in patients receiving bupivacaine. This was observed in both the THA and TKA groups (*P*≤0.001) (Table 5).

**Table 4:** Low dose (<11 mg) *vs*. High Dose (≥11 mg) bupivacaine

Outcome – *n* (%) or Avg. ± SD	Total Hip Arthroplasty			Total Knee Arthroplasty		
	High Dose Bupivacaine (***n***=213)	Low Dose Bupivacaine (***n***=222)	***P***-Value	High Dose Bupivacaine (***n***=203)	Low Dose Bupivacaine (***n***=327)	***P***-Value
Avg. Dose	12.41 ± 1.12	9.76 ± 1.23	**<0.001**	12.19 ± 1.08	9.80 ± 1.15	**<0.001**
Isobaric Bupivacaine	31 (14.6)	3 (1.4)	**<0.001**	17 (8.4)	11 (3.4)	**0.012**
0-Day LOS	48 (22.5)	56 (25.2)	0.511	18 (8.9)	41 (12.5)	0.191
LOS Days	0.95 ± 0.78	0.91 ± 0.84	0.620	1.07 ± 0.73	1.09 ± 0.98	0.823
Failed SDD Attempt	20 (6.0)	6 (6.1)	0.968	13 (3.4)	4 (2.8)	0.757
Ambulated POD0	141 (66.2)	160 (72.1)	0.185	134 (66.0)	211 (64.5)	0.728
First 6-Clicks Mobility Score^a^	20.29 ± 2.95	20.39 ± 2.66	0.740	20.11 ± 2.71	20.17 ± 2.76	0.822
First PT Ambulation Distance (ft)^b^	160.42 ± 96.02	141.07 ± 100.99	0.052	132.54 ± 100.27	133.82 ± 96.53	0.887
Urinary Retention	5 (2.3)	2 (0.9)	0.276*	2 (1.0)	4 (1.2)	1.000*
Home Discharge	206 (96.7)	214 (96.4)	0.856	195 (96.1)	318 (97.2)	0.450
30-Day Readmission	2 (0.9)	6 (2.7)	0.285*	11 (5.4)	19 (5.8)	0.850
30-Day ED Return	7 (3.3)	8 (3.6)	0.856	4 (2.0)	11 (3.4)	0.347

*P*-Values <0.05 in bold

*LOS* length of stay, *SDD* same day discharge, *POD* postoperative day, *PT* physical therapy, *ED* emergency department

*Denotes Fisher’s Exact Test

^a^n= THA 198 High, 202 Low; TKA 195 High, 307 Low

^b^n= THA 198 High, 197 Low; TKA 194 High, 307 Low

**Table 5:** Subgroup analysis of bupivacaine vs. mepivacaine in same day discharge patients

Outcome – *n* (%) or Avg. ± SD	Total Hip Arthroplasty			Total Knee Arthroplasty		
	Bupivacaine (***n***=104)	Mepivacaine (***n***=141)	***P***-Value	Bupivacaine (***n***=59)	Mepivacaine (***n***=138)	***P***-Value
Isobaric Bupivacaine	8 (7.7)	0 (0.0)	N/A	4 (6.8)	0 (0.0)	N/A
Ambulated POD0	104 (100.0)	141 (100.0)	N/A	59 (100.0)	138 (100.0)	N/A
First 6-Clicks Mobility Score^a^	21.26 ± 2.28	20.16 ± 2.38	**0.001**	22.08 ± 2.15	19.96 ± 2.54	**<0.001**
First PT Ambulation Distance (ft)^b^	193.81 ± 73.84	208.93 ± 66.60	0.144	220.25 ± 90.13	215.53 ± 82.85	0.757
Urinary Retention	1 (1.0)	2 (1.4)	1.000*	1 (1.7)	0 (0.0)	0.299*
Home Discharge	104 (100.0)	141 (100.0)	N/A	59 (100.0)	138 (100.0)	N/A
30-Day Readmission	0 (0.0)	2 (1.4)	0.509*	2 (3.4)	2 (1.4)	0.585*
30-Day ED Return	0 (0.0)	4 (2.8)	0.138*	2 (3.4)	2 (3.6)	1.000*

*P*-Values <0.05 in bold

*POD* postoperative day, *PT* physical therapy, *ED* emergency department

*Denotes Fisher’s Exact Test

^a^n= THA 91 B, 105 M; TKA 52 B, 103 M

^b^n= THA 90 B, 96 M; TKA 52 B, 81 M

### Risk-adjusted outcomes

The multivariate results presented in Table 6 display the effect of mepivacaine in comparison to bupivacaine, after controlling for age, BMI, sex, ASA, and procedure type (THA or TKA). After controlling for these factors, patients receiving mepivacaine were more likely to have 0-day LOS (OR: 5.767, *P*<0.001) and to have shorter overall LOS (β=–0.421 days, *P*<0.001). Further, they were more likely to ambulate on the day of surgery (OR: 2.391, *P*<0.001), ambulated further (β=21.785 ft., *P*<0.001), and were more likely to be discharged home (OR: 6.537, *P*=0.011). In the risk-adjusted analysis, spinal anesthetic type had no significant effect on the same day discharge failure rates, the first 6-Clicks mobility score, urinary retention, 30-day readmission, or 30-day ED returns.

**Table 6:** Risk-adjusted outcomes: bupivacaine *vs*. mepivacaine

Endpoint	Mepivacaine β/OR	95% CI	***P***-Value
0-Day LOS	5.767	4.357 – 7.634	**<0.001**
LOS Days (β)	–0.421	–0.502 – –0.339	**<0.001**
Failed SDD	0.801	0.458 – 1.400	0.436
Ambulated POD-0	2.391	1.789 – 3.197	**<0.001**
First 6-Clicks Mobility Score (β)	–0.209	–0.523 – 0.105	0.192
First PT Ambulation Distance (β)	21.785	10.459 – 33.111	**<0.001**
Urinary Retention	0.661	0.225 – 1.942	0.452
Home Discharge	6.537	1.540 – 27.743	**0.011**
30-Day Readmission	0.494	0.226 – 1.083	0.078
30-Day ED Return	1.068	0.552 – 2.067	0.845

*P*-values <0.05 in bold

*LOS* length of stay, *SDD* same day discharge, *POD* postoperative day, *PT* physical therapy, *ED* emergency department

## Discussion

In alignment with previous studies, our risk-adjusted results demonstrated that mepivacaine was associated with shorter length of stay, increased likelihood of the same-day discharge, increased early ambulation rates and distance, and increased likelihood of home discharge after both THA and TKA. However, these findings might be skewed by patient selection and preferential use of mepivacaine in patients self-selecting for early discharge, as evidenced by the lack of significant differences in outcomes when comparing bupivacaine and mepivacaine in that population. When evaluating patients receiving bupivacaine, we observed no significant differences between high and low doses. Our findings suggest that both bupivacaine and mepivacaine are safe and effective local anesthetics for patients undergoing TJA as evidenced by low, similar rates of urinary retention and 30-day ED returns and re-admissions.

The results of our study are in alignment with prior studies, demonstrating that mepivacaine use is associated with earlier ambulation, increased same-day discharge, and decreased length of stay [2, 11]. Schwenk *et al*. carried out a randomized controlled trial comparing mepivacaine (52.5 mg), hyperbaric bupivacaine (11.25 mg), and isobaric bupivacaine (12.5 mg) amongst 154 patients undergoing THA [2]. The results of their study were consistent with our data in support of mepivacaine for earlier ambulation, increased discharge rate, and shorter length of stay, although higher levels of early postoperative pain and opioid consumption occurred with mepivacaine use [2]. Additionally, the study found no significant differences in urinary retention, transient neurologic symptoms, hypotension, muscle tension or dizziness, supporting our assertion that both bupivacaine and mepivacaine are safe for use in TJA with spinal anesthesia [2]. In another retrospective review of 156 patients undergoing TKA at a single institution, mepivacaine was associated with shorter length of stay (28.1 ± 11.2 hours *vs*. 33.6 ± 14.4 hours, *P*=0.002) and less straight catheterization (3.8% *vs*. 16.5%, *P*=0.021), compared to bupivacaine. In alignment with the results of Schwenk *et al*, patients receiving mepivacaine had slightly higher pain scores and morphine consumption in the post-anesthesia care unit, but showed no difference in pain scores or morphine consumption afterwards [15]. A systematic review by Siddiqi *et al*. investigated five studies comparing mepivacaine and bupivacaine in TJA and found that mepivacaine was associated with a faster return to motor function, shorter LOS, and decreased urinary retention [16]. Further, this study found no significant differences in pain scores or ambulation distance [16]. While our study did not evaluate pain scores specifically, our finding that mepivacaine patients were more likely to ambulate POD0 and ambulate further suggests that any increased levels of early postoperative pain may not be clinically significant enough to impact early recovery. In another small randomized controlled trial comparing 32 patients receiving either bupivacaine or mepivacaine, significant differences were found in return to sensory function (*P*=0.015) and return of motor function (*P*=0.025), both favoring mepivacaine [11]. Urinary retention occurrences and time to urination were also better with mepivacaine compared to bupivacaine (*P*=0.039) [11]. A study by Calkins *et al*. evaluated spinal anesthesia in THA at an ambulatory surgery center (ASC) and found mepivacaine, when compared with bupivacaine, to be associated with less time in the ASC (*P*<0.001), decreased time to controlled voiding (*P*<0.001), and decreased time to ambulation (*P*<0.001) [17]. Further, this study found no significant differences in pain scores, complications, ED returns, or re-admissions [17]. However, bupivacaine was associated with a greater number of patients experiencing zero pain compared to the mepivacaine group [17]. Based on these studies and the results of our study, significant evidence demonstrating that mepivacaine facilitates faster ambulation and decreased length of stay exists. Our study and the Schwenk’s study deviated from the others presented in that we did not observe significant improvement in urinary retention in the mepivacaine group, although overall rates were low regardless of spinal anesthetics used. Based on prior evidence showing increased risk for postoperative urinary retention in TJA patients placed under spinal anesthesia who had a history of urinary retention and a larger volume of intraoperative fluid, we suggest restrictive intraoperative fluid management strategies be followed regardless of whether bupivacaine or mepivacaine is used [18]. Further, our results and those of Calkins *et al*. demonstrated no significant differences in 30-day ED returns or re-admissions, showing that early complications requiring intervention are similar across spinal anesthetic types. However, further study into rates of specific early and late complications is warranted to fully evaluate the effects of spinal anesthesia type on TJA recovery.

Our subgroup analysis found no significant differences between high dose and low dose bupivacaine, which is consistent with other published literature [19]. In a retrospective review of 761 TJAs, Herndon *et al*. found no significant differences in perioperative outcomes, including LOS and discharge disposition, when comparing 15 mg *vs*. <15 mg of isobaric bupivacaine [19]. While their study supports our data in finding no significant differences in surgical outcomes between high and low doses of bupivacaine, we suggest that the lowest possible therapeutic dose be used to mitigate risk of side effects.

A notable aspect of our study is the difference in unadjusted outcomes between patients undergoing THA and TKA. Specifically, 6-Clicks mobility scores and rates of home discharge showed statistically significant differences across anesthetic types in THA but not TKA patients, while 30-day re-admissions showed statistically significant differences in TKA but not THA patients. Based on the retrospective observational nature of this study, it is difficult to determine whether differences in patient characteristics and comorbidities, the surgical procedure, anesthesia type, or a combination of these factors accounts for these differences. However, our finding that mepivacaine use was not associated with differences in 6-Clicks mobility scores and re-admissions after adjusting for these confounding factors suggests that anesthesia type has less impact on these outcomes than procedure type and patient characteristics. Conversely, the finding that mepivacaine was associated with higher likelihood of home discharge after risk adjustment demonstrates that anesthesia type has a more direct effect on discharge disposition.

This study does contain multiple limitations. First, as an observational study it was exposed to selection bias, as the selection of anesthesia type was at the discretion of the provider. As previously described, our institution has informally adopted a recommendation that mepivacaine is the preferred local anesthetic for planned same-day discharge TJAs. This therefore makes it difficult to decipher whether the favorable outcomes observed in the mepivacaine group were due to patient selection or the anesthetic itself. Second, it is possible that additional confounding factors, including other specific comorbidities or psychosocial factors, influenced the observed outcomes. Third, our evaluation of isobaric and hyperbaric bupivacaine preparations was underpowered, thus limiting any conclusions that can be drawn from any negative results.

## Conclusion

The results of this study demonstrated that both bupivacaine and mepivacaine are safe and effective local anesthetics for patients undergoing TJA with spinal anesthesia. However, mepivacaine is associated with shorter length of stay, increased likelihood of the same-day discharge, increased early ambulation rates and distance, and increased likelihood of home discharge after both THA and TKA. We therefore suggest that mepivacaine should be considered the first-choice spinal anesthetic for patients undergoing rapid recovery TJA.

## Data Availability

The datasets used and/or analyzed during the current study are available from the corresponding author on reasonable request.

## Abbreviations

SA: Spinal anesthesia
TJA: Total joint arthroplasty
M: Mepivacaine
HB: Hyperbaric bupivacaine
IB: Isobaric bupivacaine
SDD: Same-day-discharge
CCI: Charlson comorbidity index
THA: Total hip arthroplasty
TKA: Total knee arthroplasty
LOS: Length of stay
DVT: Deep venous thrombosis
RA: Regional anesthesia
ASC: Ambulatory surgery center
BMI: Body mass index
ASA: American Society of Anesthesiologists
POD0: Postoperative day 0
AM-PAC: Activity measure for post-acute care
PT: Physical therapy
